# Embryonal Sarcoma of the Liver in an Adult Patient

**DOI:** 10.1155/2012/382723

**Published:** 2012-05-29

**Authors:** Nicole Lightfoot, Mehrdad Nikfarjam

**Affiliations:** ^1^Department of Pathology, The University of Melbourne, Austin Health, Heidelberg, Melbourne, VIC 3084, Australia; ^2^Department of Surgery, Austin Health, The University of Melbourne, Heidelberg, Melbourne, VIC 3084, Australia

## Abstract

Undifferentiated embryonal sarcomas (UESs) are uncommon tumours that are seen predominantly in late childhood. Cases in adults are rare and generally present once a large mass develops and may be mistaken for other tumours. A case of an UES of the liver with an isolated peritoneal metastasis is described. The patient presented with a palpable mass with imaging findings suggestive of a cystic tumour. She had complete surgical resection of the liver mass and isolated peritoneal metastasis. She was tumour-free on imaging at 6 months without adjuvant chemotherapy. An UES should be considered in the differential of large cystic hepatic lesions, with aggressive surgical resection considered when possible.

## 1. Introduction

Undifferentiated embryonal sarcoma (UES) is an extremely rare entity in adulthood, with less than 60 cases reported in the literature [1–4]. Embryonal sarcoma more typically occurs in children, with a peak incidence between the ages of 6 and 10 years [5]. Embryonal sarcoma represents the third most common primary pediatric liver tumour after hepatoblastoma and hepatocellular carcinoma.

The behavior of UES is generally highly aggressive in children and is considered to be the same in adults. Preoperative diagnosis of UES is rarely entertained for adults. Surgical resection does offer a chance of possible cure and should be considered in all cases. A case of UES in an adult patient treated by aggressive surgery is presented, and the features of UES and outcomes are discussed.

## 2. Case

**Figure 1:**
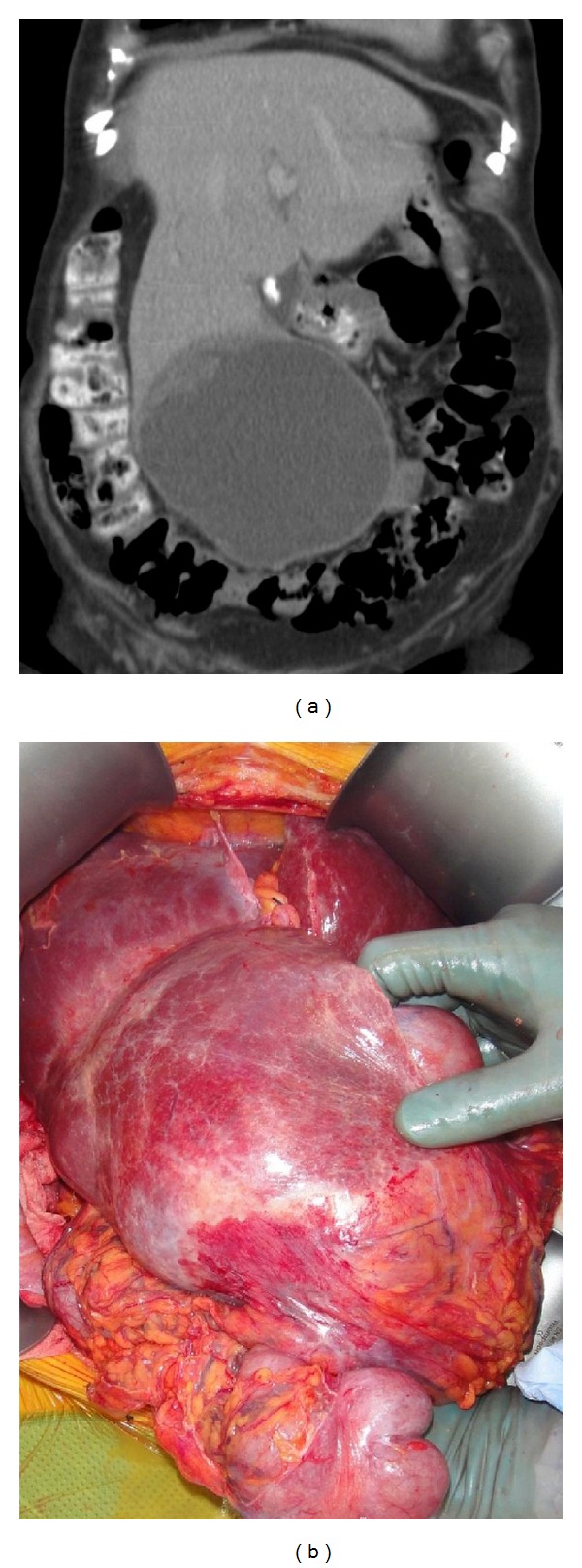
(a) Coronal computed tomography imaging demonstrating cystic tumour within the liver with more peripheral solid component. (b) Intraoperative image of large, partially haemorrhagic hepatic tumour overlying the right colon.

A 78-year-old female presented with asymptomatic upper abdominal mass. Computed tomography (CT) was performed and demonstrated an encapsulated solid and cystic tumour involving the right lobe of the liver, measuring 16 cm in maximum diameter (Figure 1(a)). Positron emission tomography (PET) showed intense peripheral metabolic activity with a photopenic core, suspicious for a malignant tumour with central cystic, haemorrhagic or necrotic change. There was no evidence of underlying liver disease on blood tests, and tumour markers including carcinoembryonic antigen (CEA), carbohydrate antigen (CA-19.9), alpha feto protein (AFP), and chromogranin A were within normal limits. 

At the time of surgery a large, a well-circumscribed, partially haemorrhagic cystic lesion was noted (Figure 1(b)). This was adherent to the proximal colon and omentum, however, with no clear direct invasion. A 3 cm cystic nodule was also noted in the right pelvis. A partial right hepatectomy and en bloc extended right hemicolectomy were performed as well as removal of the pelvic nodule. The patient's post-operative course was unremarkable. 

**Figure 2:**
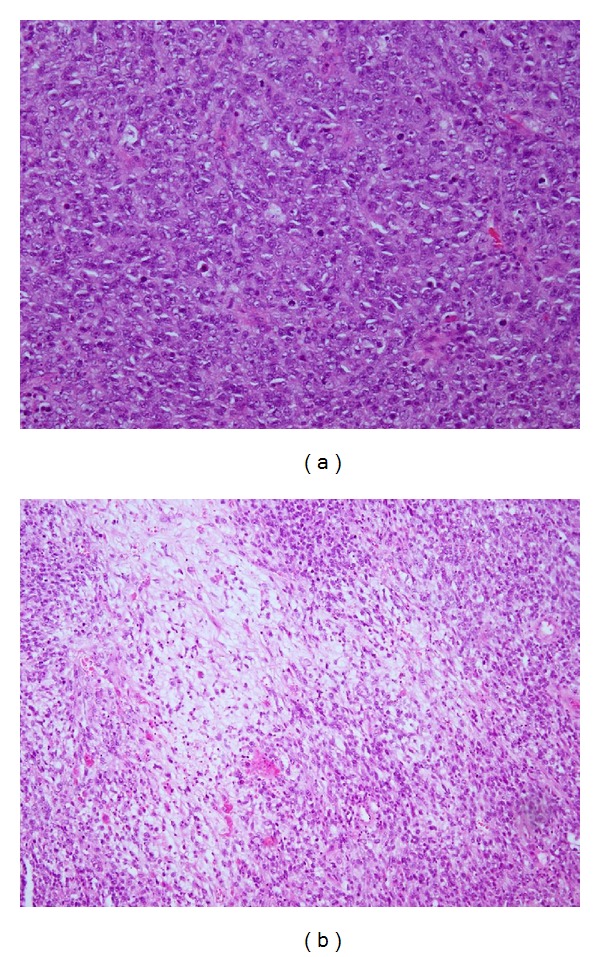
(a) Microscopic view of densely cellular area comprised of pleomorphic round and spindled cells (hematoxylin and eosin, ×200). (b) Paucicellular area with myxoid matrix (hematoxylin and eosin, ×200).

**Figure 3:**
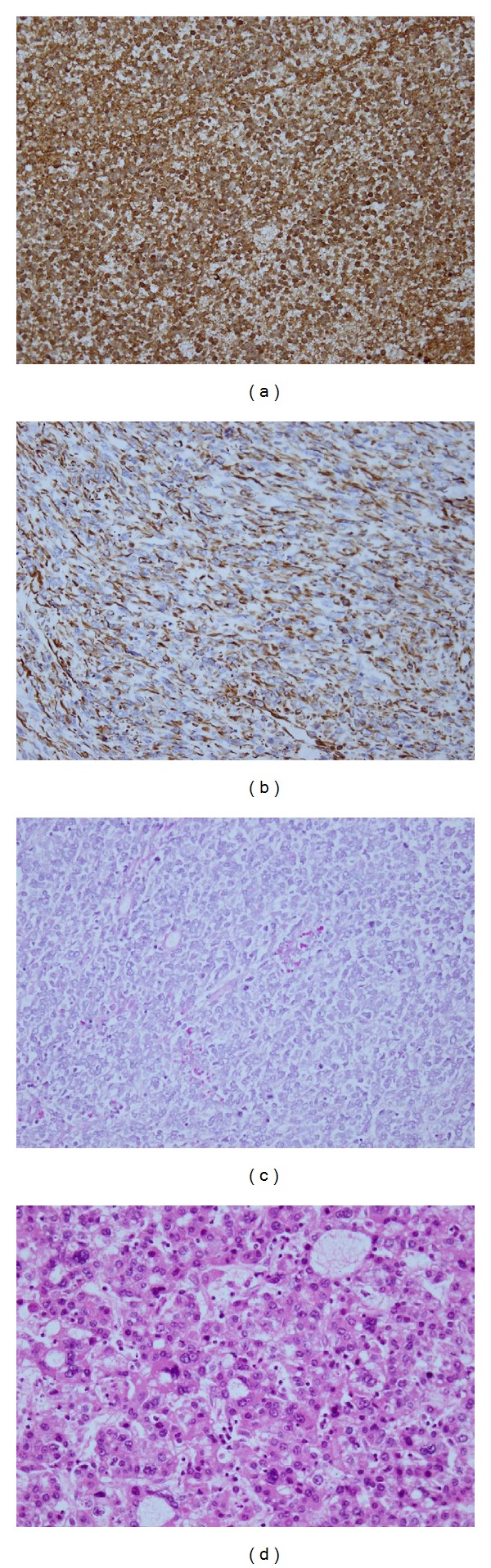
(a) Alpha_1_-antitrypsin immunohistochemistry diffusely staining tumour cells (×200). (b) Vimentin immunohistochemistry staining tumour cells (×200). (c) PAS-positive, diastase-resistant intratumoral cytoplasmic and extracellular eosinophilic globules (×200). (d) Malignant hepatoid tumour nodule within the tumour (hematoxylin and eosin, ×200).

Histology of the tumour revealed variable morphology. Prominent intratumoral necrosis was seen, and there was moderate mitotic activity. The morphological and immunohistochemical profile was consistent with undifferentiated embryonal sarcoma. The tumour was comprised of densely cellular areas of rounded and pleomorphic cells and myxoid more paucicellular areas with stellate and spindled cells (Figure 2). Tumour cells stained positively for AE1/AE3, alpha_1_-antitrypsin, vimentin, and desmin. Variably sized eosinophilic, intracytoplasmic, PAS-positive, diastase resistant globules were also noted within occasional tumour cells (Figure 3). In addition, nodules of malignant hepatoid tumour were admixed with the undifferentiated areas (Figure 3(d)) and stained positively for HepPar-1, polyclonal carcinomembryonic antigen, and AE1/AE3.

The patient declined adjuvant chemotherapy. Repeat CT imaging 6 months postoperatively showed no evidence of recurrent disease. 

## 3. Discussion

Embryonal sarcoma is a primitive mesenchymal tumour of unknown aetiology associated with multiple genetic mutations [6]. Also known as malignant mesenchymoma, mesenchymal sarcoma, primary sarcoma, undifferentiated sarcoma, fibromyxosarcoma, and rhabdomyosarcoma of the liver, UES is a highly aggressive tumour with a generally poor prognosis.

UES most commonly arises in the right lobe of the liver; however cases located within the left lobe and bilateral lobes simultaneously have been documented [4, 7]. Variable gender predilection is reported, ranging from an equal gender incidence to a slight female predominance in the adult population [4, 7, 8].

Preoperative diagnosis is confounded by the absence and/or nonspecific symptoms. Embryonal sarcoma may present with abdominal pain or palpable mass or represent an incidental radiological finding as in our case. Tumoral rupture may rarely result in intraperitoneal haemorrhage [7]. Constitutional symptoms of anorexia, weight loss, and fevers are also reported [1, 9]. Tumour antigens are of limited utility diagnostically as serum alpha feta protein may be elevated or more typically within normal limits [6, 10]. Mild liver function derangement has been reported in some cases [4]; however no association with cirrhosis or hepatitis has been demonstrated [1].

Radiologically, UES presents as a solid, cystic, or complicated circumscribed mass with or without septations [1]. CT may show areas of haemorrhage or necrosis; however the ultrasonographic appearance is variable. A uniform finding from all imaging modalities is the large tumour size, ranging from 10 to 35 cm [2, 4]. Pre-operative imaging accordingly generates a broad list of differential diagnoses. These include primary hepatic cystic neoplasms such as biliary cystadenoma, biliary cystadenocarcinoma, and cavernous haemangioma; infectious aetiologies such as hydatid cyst and amoebic abscess; cystic degeneration within a primary liver tumour or the atypical appearance of a metastasis. Additional worthwhile investigations to narrow the diagnostic spectrum preoperatively include hydatid serology and PET scan. The role of fine needle aspiration is equivocal and should be undertaken with caution based on the documented case of UES seeding along a surgical drain site [3]. UES has been presumptively diagnosed as metastatic ovarian serous papillary carcinoma and acute appendicitis, respectively, amongst its myriad presentations [11, 12].

Macroscopic examination of UES reveals a well-circumscribed, soft mass showing cystic degeneration, haemorrhagic, and necrotic areas. Microscopically the tumour is surrounded by a pseudocapsule often with foci of extracapsular infiltration. UES is typically composed of stellate and spindled cells, set within a myxoid matrix and areas of higher cellularity. Moderate nuclear hyperchromasia and pleomorphism are seen. Occasional bizarre giant cells and PAS-positive, diastase-resistant eosinophilic globules are typically within the cytoplasm [13]. Extramedullary haematopoiesis is often noted. Tumour cells are generally positive for vimentin and alpha_1_-antitrypsin immunohistochemical stains, with variable cytokeratin expression.

UES is a highly aggressive neoplasm with a guarded prognosis. Complete surgical resection offers the only chance of long-term survival, further improved by adjuvant chemotherapy plus or minus radiotherapy. While patients have been documented as disease-free for greater than 10 years, the median survival is reported as 29 months [14]. Poor prognosis is associated with unresectability, positive surgical margins-and spontaneous or iatrogenic tumour rupture [2]. Common sites for metastases include lung, pleura, and peritoneum. Tumour may also show direct involvement of the heart, with inferior vena cava tumour extension to the right atria. Liver transplantation has been performed in the pediatric population for UES; however to date no adult has undergone this treatment [3].

In summary, UES of liver in the adult population is a very rare entity with limited cases reported. When tumours are asymptomatic and the radiological features nonspecific, the diagnosis of embryonal sarcoma preoperatively is speculative and many other differentials are feasible. Surgical resection is necessary for histological assessment and complete tumour removal. Embryonal sarcomas have generally poor prognosis; however surgical resection and adjuvant chemoradiation offer the best chance for long-term survival.
